# Fatty acid mimetic PBI-4547 restores metabolic homeostasis via GPR84 in mice with non-alcoholic fatty liver disease

**DOI:** 10.1038/s41598-020-69675-8

**Published:** 2020-07-29

**Authors:** Jean-Christophe Simard, Jean-François Thibodeau, Martin Leduc, Mikael Tremblay, Alexandre Laverdure, François Sarra-Bournet, William Gagnon, Jugurtha Ouboudinar, Liette Gervais, Alexandra Felton, Sylvie Letourneau, Lilianne Geerts, Marie-Pier Cloutier, Kathy Hince, Ramon Corpuz, Alexandra Blais, Vanessa Marques Quintela, Jean-Simon Duceppe, Shaun D. Abbott, Amélie Blais, Boulos Zacharie, Pierre Laurin, Steven R. Laplante, Christopher R. J. Kennedy, Richard L. Hébert, François A. Leblond, Brigitte Grouix, Lyne Gagnon

**Affiliations:** 1 Liminal R&D Biosciences Inc., 500 Boulevard Cartier Ouest (Suite 150), Laval, QC H7V 5B7 Canada; 2 Department of Cellular and Molecular Medicine, Kidney Research Centre, Ottawa Hospital Research Institute, University of Ottawa, 451 Smyth Road, Ottawa, ON K1H 8M5 Canada; 3 Institut National de La Recherche Scientifique, Institut Armand-Frappier, 531 Boul. Des Prairies, Laval, QC H7V 5B7 Canada

**Keywords:** Cell biology, Drug discovery, Molecular biology, Physiology, Diseases, Gastroenterology

## Abstract

Non-alcoholic Fatty Liver Disease (NAFLD) is the most common form of liver disease and is associated with metabolic dysregulation. Although G protein-coupled receptor 84 (GPR84) has been associated with inflammation, its role in metabolic regulation remains elusive. The aim of our study was to evaluate the potential of PBI-4547 for the treatment of NAFLD and to validate the role of its main target receptor, GPR84. We report that PBI-4547 is a fatty acid mimetic, acting concomitantly as a GPR84 antagonist and GPR40/GPR120 agonist. In a mouse model of diet-induced obesity, PBI-4547 treatment improved metabolic dysregulation, reduced hepatic steatosis, ballooning and NAFLD score. PBI-4547 stimulated fatty acid oxidation and induced gene expression of mitochondrial uncoupling proteins in the liver. Liver metabolomics revealed that PBI-4547 improved metabolic dysregulation induced by a high-fat diet regimen. In *Gpr84*^*−/−*^ mice, PBI-4547 treatment failed to improve various key NAFLD-associated parameters, as was observed in wildtype littermates. Taken together, these results highlight a detrimental role for the GPR84 receptor in the context of meta-inflammation and suggest that GPR84 antagonism via PBI-4547 may reflect a novel treatment approach for NAFLD and its related complications.

## Introduction

Non-Alcoholic Fatty Liver Disease (NAFLD) is the most prevalent chronic liver disease worldwide and is among the top three causes of liver transplantation in the US. NAFLD represents the pathological hepatic manifestation of metabolic dysregulation including fatty acid (FA) metabolism, not caused by excessive alcohol consumption, pro-steatogenic drugs or hereditary disorders and is defined by the presence of steatosis in the liver, due to triglyceride accumulation in hepatocytes (> 5% of total fat content)^1^. Pathological signs range from steatosis to non-alcoholic steatohepatitis (NASH), which represents the most severe condition. Although the precise molecular mechanisms underlying the progression from simple steatosis to NASH remain elusive, several key risk factors are known to be intimately involved including inflammation, diet and infectious agents. The most important risk factor for NAFLD/NASH is type-II diabetes mellitus and remains a strong predictor for development of adverse clinical outcomes including advanced liver fibrosis and death. Other aggravating factors, such as fibrosis in itself^2^ are also intimately linked to the progression of NAFLD and targeting this has shown to be important in the treatment of this disease^3^.

Mitochondria are the intracellular organelles responsible for ATP generation mainly through utilization of glucose and FAs. Mitochondrial dysfunction, often associated with enhanced mitochondrial reactive oxygen species (ROS) production and defective FA oxidation (FAO), is present in liver samples from patients with NAFLD and diabetes (reviewed in Ref.^4^. FAO is mainly regulated by CD36, one of the scavenger receptors responsible for the uptake of FFA, while FA transport inside the mitochondria is regulated by carnitine palmitoyltransferase 1 (CPT1). Both cellular uptake and mitochondrial translocation are now considered the rate limiting steps in FA metabolism. Besides their effects on intracellular metabolism and nuclear receptors, FFAs can activate distinct cell surface G protein-coupled receptors, including FFA receptors 1 and 4 (also known as GPR40 and GPR120, respectively) and GPR84. GPR40 and GPR120 are activated by both medium- and long-chain FFAs, while GPR84 is exclusively responsive to medium-chain FFAs^5^. FA binding to GPR40 on pancreatic β-cells leads to activation of several signaling pathways involved in insulin secretion and targeting this receptor has shown to be a promising new treatment for T2DM, however safety concerns (hepatotoxicity) associated with current lead compounds have precluded their clinical use^6^. Recently, a dual GPR40 and GPR120 agonist showed potent activity on both adipose tissue lipolysis and glucose metabolism, highlighting the strong potential of these receptors in FA and glucose metabolism^7^.

We have reported that our lead drug candidate currently in clinical testing, PBI-4050, exerts its anti-fibrotic effects in models of kidney, heart, liver, lung, pancreas, and skin fibrosis via concomitant activation of GPR40 and inhibition of GPR84^8^. GPR84 is expressed in monocytes, neutrophils and macrophages and is induced under pro-inflammatory stimuli. The gene sequence for GPR84 is not closely related with other GPCRs but is highly conserved in rodents and humans (85% sequence identity). To date, little information is available regarding the role of GPR84 in humans. Most of our understanding of this receptor has been derived from studies using primary human cells. Accordingly, it’s been demonstrated that GPR84 activation using a pharmacological agonist (embelin) induces chemotaxis and primes the neutrophil oxidative burst^9^. The notion that GPR84 is involved in both immune and metabolic regulation has been described, albeit incompletely. For instance, it’s been shown that in human fat cells derived from pre-adipocytes, GPR84 expression levels are induced by pro-inflammatory signals, including TNF-α, IL-1β^10^ and IL-33, a member of the IL-1β superfamily^11^. According to available datasets, adipose tissue GPR84 expression is higher in morbidly obese compared to non-obese individuals (NCBI GEO Profiles, accession GDS5056), while adipose tissue from diabetics have higher GPR84 levels in CD14 + monocytes than non-diabetics (accession GDS5167).

An advanced screening program for new GPCR-modulating compounds identified PBI-4547 as a potential therapeutic candidate for the treatment of T2DM-associated metabolic syndrome and NAFLD. In this study, we report the beneficial effects of this second-generation fatty acid modulator, PBI-4547, on NAFLD pathogenesis and on regulation of glucose and FA metabolism in a model of diet-induced obesity/metabolic syndrome. Furthermore, using knockout mice, we identify the pro-inflammatory GPR84 receptor as a key player in metabolic dysregulation, and as a primary target of PBI-4547 in the context of obesity-related metabolic syndrome.

## Results

### PBI-4547 is an agonist of GPR40/120 and an antagonist of GPR84

**Figure 1:**
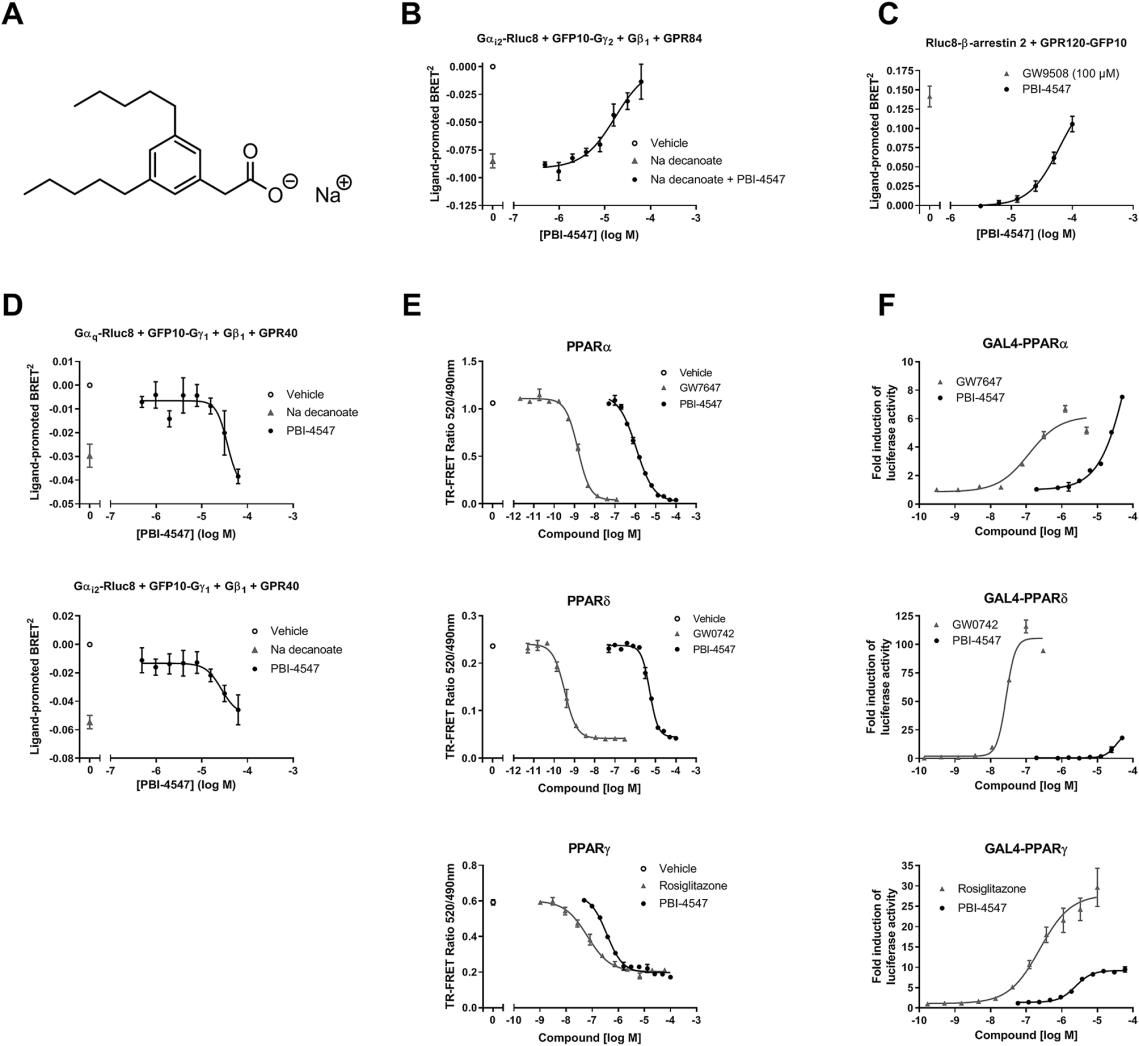
PBI-4547 binding and activity on GPR84, GPR40, GPR120, and PPAR receptors. (**A**) Chemical structure of PBI-4547. (**B**) G_i_ pathway activation was monitored in HEK293 cells transfected with GPR84 and the Gα_i2_ activation biosensor. Cells were co-treated with 125 mM sodium decanoate and increasing concentrations of PBI-4547, and BRET variation compared with vehicle was measured. (**C**) β-arrestin 2 recruitment to activated GPR120 receptor was monitored in HEK293 cells transfected with RLuc8-β-arrestin 2 and GPR120-GFP10. (**D**) Cells transfected with GPR40 and Gα_q_ or Gα_i2_ activation biosensor were exposed to increasing concentrations of PBI-4547 or to 500 µM of sodium decanoate, and BRET variation compared with vehicle was measured. Cells were exposed to increasing concentrations of PBI-4547 or to 100 µM of GW9508, and BRET variation compared with vehicle was measured. (**E**) LanthaScreen TR-FRET competitive binding assay of PBI-4547 and PPAR control agonists to the LBD of PPARα, PPARδ and PPARγ. (**F**) Cell-based GAL4 transactivation assay in HEK293 cells transfected with the LBD of PPARα, PPARδ or PPARγ, and treated with increasing concentrations of PBI-4547 or PPAR control agonists. Data in (**B**–**D**) are expressed as means ± SEM of 4–5 experiments; data in (**E,F**) are from a representative experiment from a total of 3 to 4 independent experiments.

PBI-4547 (2-[3,5-dipentylphenyl]acetate sodium salt) is a medium-chain FA (MCFA) mimetic (Fig. 1A). Accordingly, we examined the signaling properties of PBI-4547 on receptors known to be activated by MCFAs. GPR84 couples to the Gα_i/o_ family of G proteins and hence, a previously described bioluminescence resonance energy transfer (BRET) biosensor^8^ was used to directly monitor Gα_i_ protein activation in living HEK293 cells. This biosensor consisted of an Rluc8-tagged Gα_i2_ subunit, a GFP10-tagged Gγ_2_ subunit, and an untagged Gβ_1_. Agonist stimulation and receptor activation triggers a physical separation between the Gα-Rluc8 donor and the GFP10-Gγ_2_ acceptor, resulting in a decrease in BRET signal, whose amplitude is correlated to ligand efficacy. Indeed, treatment with the GPR84 agonist sodium decanoate induced a BRET signal decrease compared to vehicle-treated cells (Fig. 1B). Treatment with PBI-4547 did not lead to GPR84/Gα_i2_-mediated BRET signal modulation (results not shown). However, co-treatment with sodium decanoate and increasing concentrations of PBI-4547 led to a concentration-dependent inhibition of GPR84/Gα_i2_ activation (reflected by an increase in BRET signal) with a half maximal inhibitory concentration (IC_50_) of 17 µM.

G-protein BRET biosensors were also used to monitor PBI-4547 dependent activation of GPR40, which has been described as coupling mainly to the Gα protein subunit of the G_q_ family, and partially to G_i_^12^. Treatment with increasing concentrations of PBI-4547 resulted in a BRET signal decrease for both Gα_q_ (half maximal effective concentration (EC_50_): 30 µM) and Gα_i2_ (EC_50_: 27 µM) biosensors (Fig. 1D), indicative of GPR40 binding and activation with similar potency profile as previously reported for sodium decanoate for these signaling effectors (EC_50_ of 102 µM for Gα_q_ and 16 µM for Gα_i2_)^8^.

Upon agonist binding, GPR120 interacts robustly with β-arrestins 1 and 2, and therefore we assessed PBI-4547′s agonistic activity towards GPR120 by employing a BRET-based assay that allows the monitoring of Rluc8-tagged β-arrestin-2 recruitment to GFP10-tagged GPR120. We found that PBI-4547 dose-dependently promoted β-arrestin-2 recruitment to GPR120, with an EC_50_ of 48 µM (Fig. 1C).

### PBI-4547 is a partial ligand and activator of PPARs

The peroxisome proliferator-activated receptors (PPARs), PPARα, PPARδ, and PPARγ are ligand-dependent transcription factors regulated by FAs, including MCFAs, which control expression of several key metabolism-associated genes. We thus characterized the binding and transcriptional activity of PBI-4547 to these receptors. A LanthaScreen TR-FRET competitive binding assay was used to determine the binding affinity of PBI-4547 in comparison to the full control agonists GW7647 (PPARα), GW0742 (PPARδ), and rosiglitazone (PPARγ). Binding of PBI-4547 to the three PPAR ligand binding domains (LBDs) was observed, although with lower affinity than the control agonists, with affinity for PPARγ > PPARα > PPARδ, and K_i_ inhibition constants of 0.088, 0.489, and 3.16 µM, respectively (Fig. 1E).

To determine the transcriptional activity of PBI-4547, a cell-based GAL4 transactivation assay in HEK293 cells transfected with either PPARα, PPARδ, or PPARγ LBD was used. In agreement with the binding assay, the potency of PBI-4547 to drive transcriptional activity was stronger for PPARγ (EC_50_ of 3.6 µM) than PPARα (EC_50_ of 42 µM) (Fig. 1F). However, PBI-4547 only partially activated PPARγ transcriptional activity, with a relative maximal efficacy of 38% of the control agonist rosiglitazone. Finally, when compared to the control agonist GW0742, PBI-4547 showed very weak PPARδ transcriptional activity.

### PBI-4547 improves glucose metabolism in a mouse model of NAFLD

**Figure 2:**
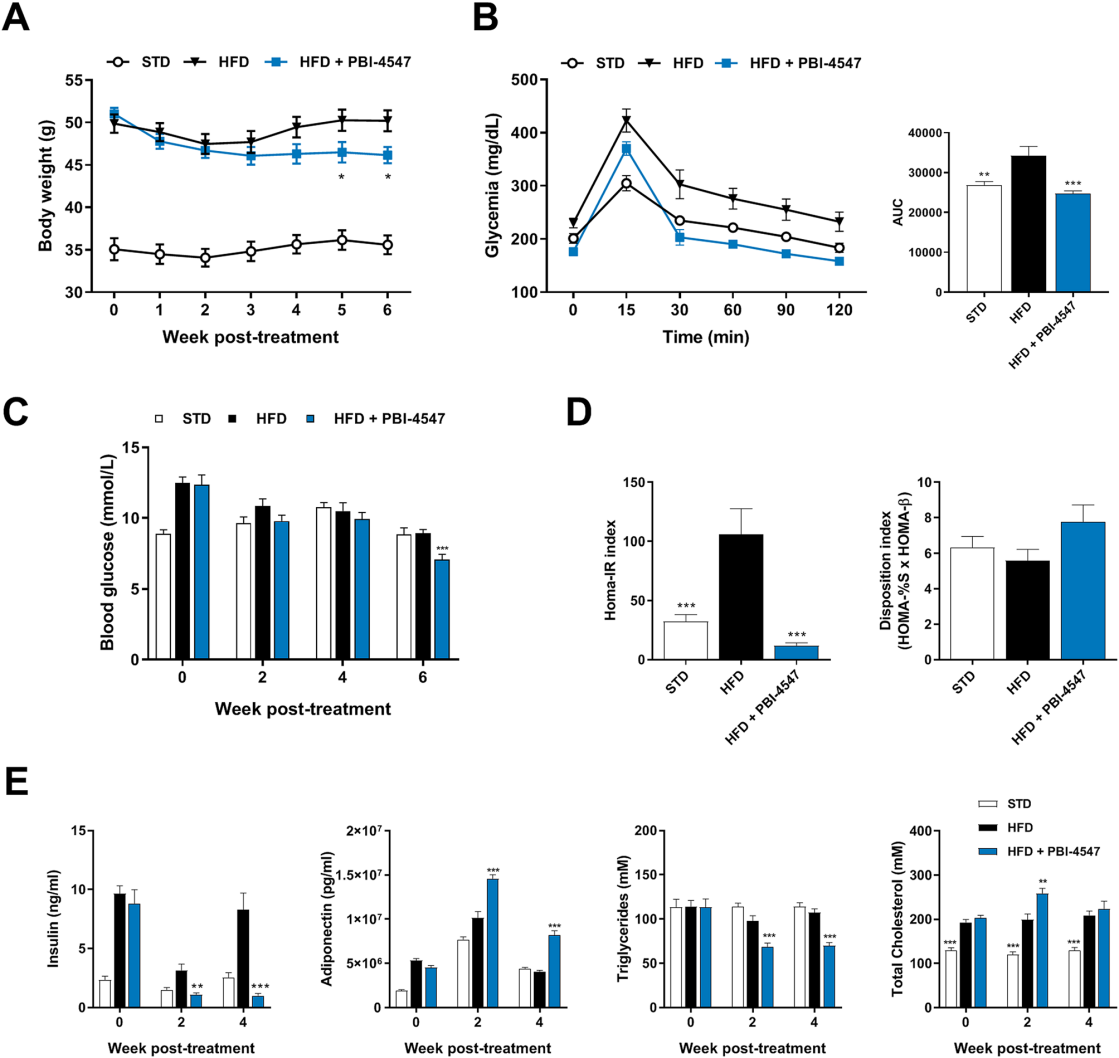
PBI-4547 improves serum parameters and insulin resistance in HFD mice. (**A**) Average body weight of STD, HFD and HFD + PBI-4547 mice. (**B**) Oral Glucose Tolerance Test (OGTT) and corresponding area under the curve after 4 weeks of treatment. (**C**) Average fasting blood glucose of the mice over 6 weeks of treatment.(**D**) Insulin resistance (HOMA-IR) and glucose disposition index of mice after 4 weeks of treatment. (**E**) Quantification of serum parameters: Insulin, adiponectin, triglycerides and total cholesterol in serum of mice after 0, 2 and 4 weeks of treatment. Data are presented as mean ± SEM (n ≥ 6 per group, one-way ANOVA with Dunnett’s multicomparison test vs HFD group). See also Fig. S1.

The well-established high-fat diet (HFD) mouse model of obesity was used to study the impact of PBI-4547 on the onset and progression of NAFLD. A 14-week HFD regimen led to significant weight gain in both male and female mice. Importantly, PBI-4547 led to a significant reduction of body weight in mice fed a HFD 5 weeks after the beginning of treatment (Fig. 2A) without affecting food intake or organ weight (Fig. S1). Fasting blood glucose was decreased by PBI-4547 after 6 weeks of treatment (Fig. 2B), while an oral glucose tolerance test (OGTT) showed that mice treated with PBI-4547 had better glycemic control (Fig. 2C). Although the disposition index remained unchanged, the HOMA-IR index, a measure of insulin-resistance, was decreased by PBI-4547 to the STD group level, suggesting improved insulin sensitivity (Fig. 2D). After 2 weeks of drug treatment, fasting insulin levels in all groups were lower than at baseline, however HFD-fed vehicle treated mice had significantly elevated insulin levels compared to both standard diet and PBI-4547-treated groups, (Fig. 2E), highlighting a potential insulin-sensitizing effect with this compound. Moreover, PBI-4547 decreased serum triglycerides while enhancing adiponectin levels. Finally, total cholesterol was slightly increased by PBI-4547 after two weeks of treatment but returned to similar level as in the HFD group after four weeks. Of note, the effects of PBI-4547 on bodyweight, food consumption, glucose tolerance, fasting blood glucose, insulin resistance and HOMA-β indices were absent in standard diet-fed mice (Table S1).

### PBI-4547 prevents progression of NAFLD

**Figure 3:**
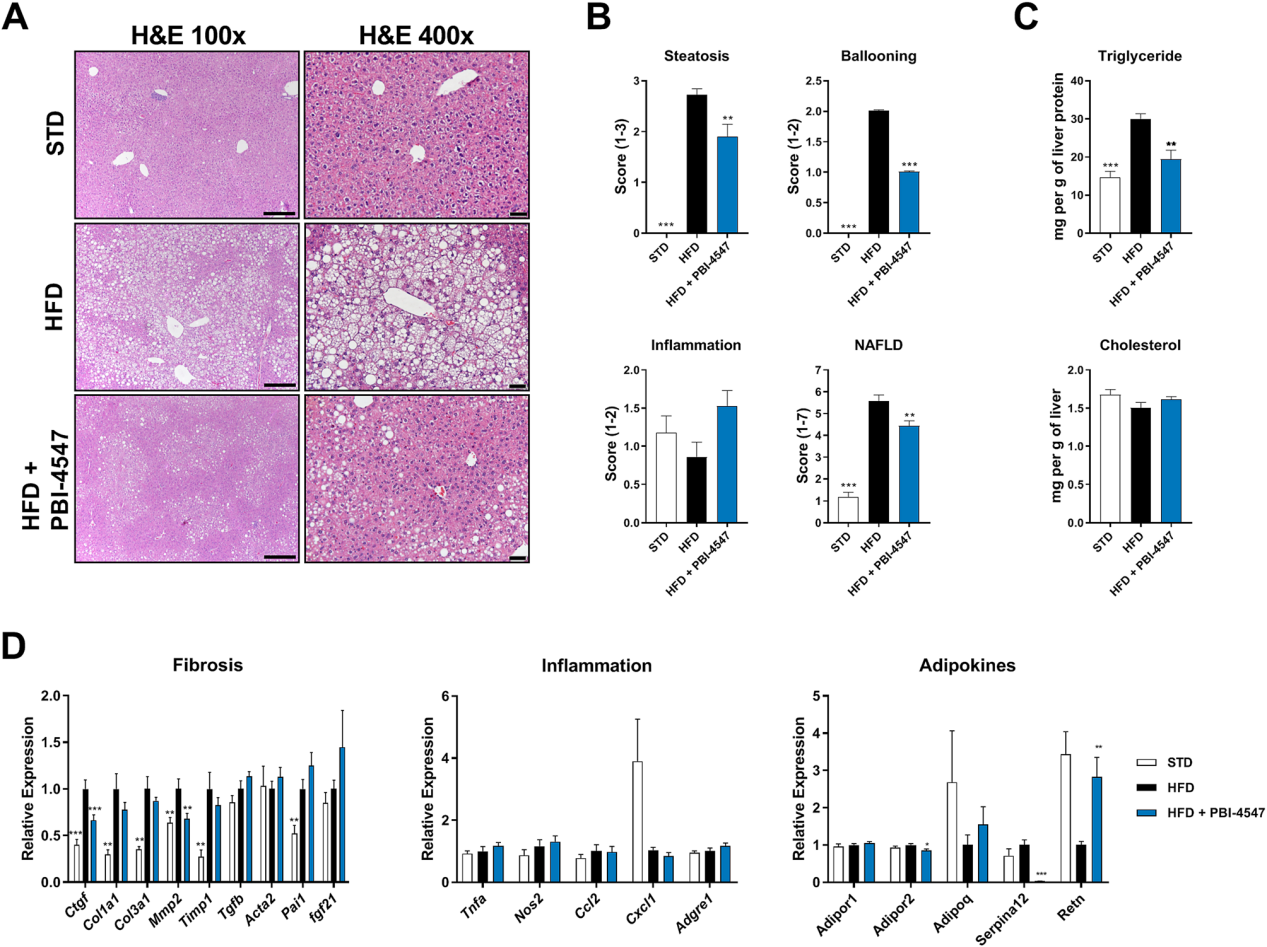
Effects of PBI-4547 intervention on NAFLD development. (**A**) Representative image of HE-stained liver section of STD, HFD and HFD + PBI-4547 mice. (**B**) Scoring of steatosis, ballooning, inflammation and total NAFLD score in liver sections of the mice. (**C**) Quantification of hepatic triglyceride and cholesterol levels. (**D**) Relative expression levels of genes involved in fibrosis, inflammation and with adipokine. Mean expression of qRT-PCR data was set to 1 for HFD group. Data are presented as mean ± SEM (n ≥ 6 per group, one-way ANOVA with Dunnett’s multicomparison test vs HFD group).

Histological assessment revealed that treatment with PBI-4547 halted the establishment of severe NAFLD due to HFD-feeding, as compared to vehicle-treated mice (Fig. 3A). Although the degree of hepatic inflammation remained unchanged by diet or drug-treatment, steatosis and ballooning were common findings in HFD-fed mice, but were markedly reduced by PBI-4547 treatment, significantly improving the overall NAFLD score (Fig. 3B). Moreover, PBI-4547 reduced liver triglyceride levels without affecting levels of total cholesterol (Fig. 3C).

Since fibrosis contributes directly to the progression from NAFLD to NASH, we next evaluated the effect of PBI-4547 pro-fibrotic gene expression. Accordingly, mRNA levels of *Ctgf* and *Mmp2* were significantly decreased by PBI-4547 (Fig. 3D). Pro-inflammatory genes were not modulated by treatment with PBI-4547. However, adipokine-related genes, *Adipor2* and *Serpina12* (vaspin), were downregulated by PBI-4547 while *Retn* (resistin) was restored to normal levels. As liver fibrosis was relatively mild in HFD-fed mice, we performed separate studies to evaluate the antifibrotic effect of PBI-4547, in rodent models of carbon tetrachloride (CCl_4_)- and bile duct ligation (BDL)-induced liver fibrosis. Compared to control animals, CCl_4_ mouse and BDL rat livers showed marked collagen deposition, which was significantly decreased by PBI-4547 (Fig. S2). Collectively, these results indicate that PBI-4547 effectively reduces several clinical manifestations of NAFLD, including fibrosis.

### PBI-4547 restores hepatic glucose and FA metabolism

**Figure 4:**
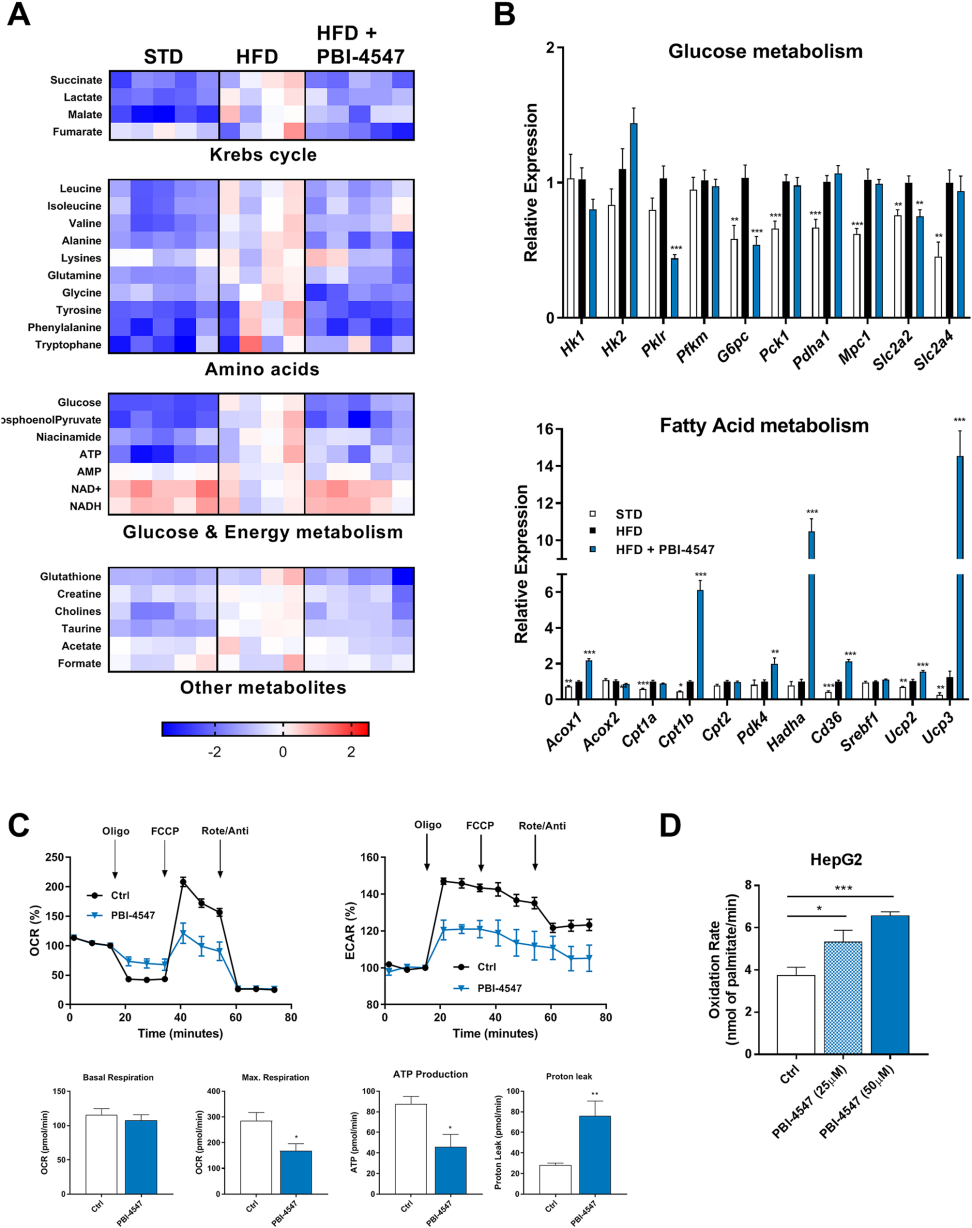
Effects of PBI-4547 on glucose and FA metabolism in liver. (**A**) Relative gene expression levels of glucose and FA-related metabolism in liver from STD, HFD and HFD + PBI-4547 mice. Data are presented as geometric mean ± SEM (n ≥ 6 per group, one-way ANOVA with Dunnett’s multicomparison test vs HFD group). (**B**) 1H-NMR metabolic profiling in liver tissues. (**C**) Oxygen Consumption Rate (OCR) and Extracellular Acidifcation Rate (ECAR) in HepG2 hepatocytes. Measurements of basal respiration, maximal respiration, ATP production and proton leak in HepG2 hepatocytes treated or not with PBI-4547. (**D**) Oxidation rate of ^14^C-palmitate in HepG2 hepatocytes treated or not with PBI-4547. Data are presented as mean ± SEM (n = 3, Student t test Ctrl vs PBI-4547). See also Fig. S2.

Metabolomics analysis revealed severe hepatic dysregulation in the metabolism of key amino acids and citric acid cycle intermediates in HFD-fed mice, which was corrected by PBI-4547 treatment (Fig. 4A and Fig. S3). Energy metabolism intermediates were also affected in HFD setting and restored back to normal levels by PBI-4547. Moreover, hepatic expression of several genes involved in glucose metabolism including *Pklr* (pyruvate kinase L/R), *G6pc* (glucose-6-phosphatase, catalytic) and *Slc2a2* (Glut2) was normalized by PBI-4547 (Fig. 4B). Additionally, *Ucp2* and *Ucp3* expression were highly upregulated by PBI-4547, suggesting an uncoupling activity of this compound. In accordance with these results, we observed a strong upregulation of several genes involved in FA metabolism, including *Acox1* (acyl-CoA oxidase 1)*, Cpt1b* (carnitine palmitoyltransferase 1B)*, Pdk4* (pyruvate dehydrogenase kinase 4), *Hadha* (hydroxyacyl-CoA dehydrogenase) and *Cd36*, suggesting that PBI-4547 could affect mitochondrial dynamics. To test this hypothesis, mitochondrial respiration in HepG2 hepatocytes was measured (Fig. 4C). PBI-4547 did not affect basal oxygen consumption rate (OCR). However, it did decrease maximal respiration and ATP production, in agreement with the metabolomics results. In accordance with increased levels of *Ucp2/3*, mitochondrial proton leak was also significantly increased by PBI-4547. Next, to verify the direct impact on FAO, we incubated HepG2 hepatocytes with ^14^C-palmitate to measure β-oxidation rate and found that PBI-4547 dose-dependently increased oxidation of palmitate (Fig. 4D).

**Figure 5:**
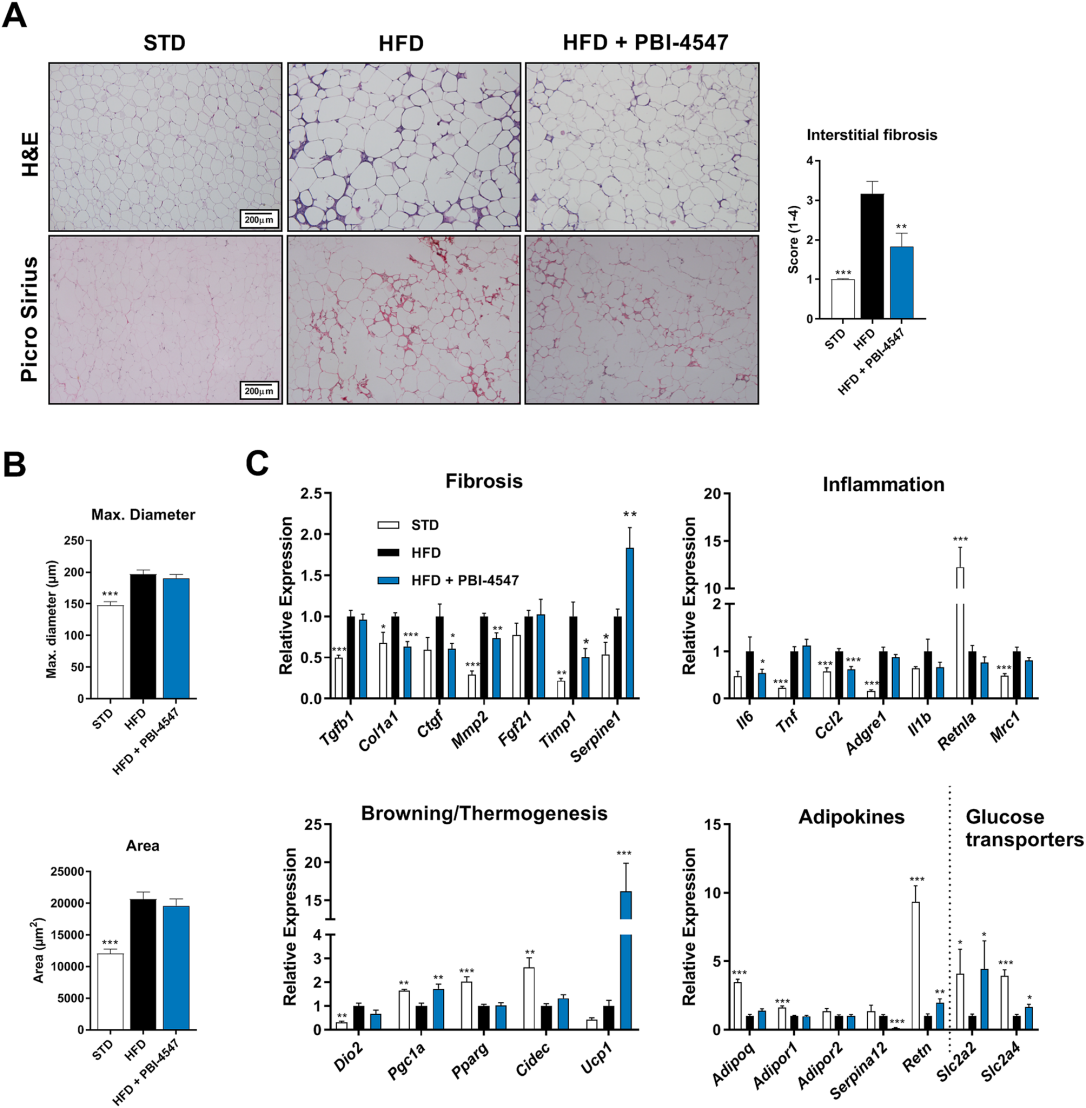
Effects of PBI-4547 intervention on WATs in a HFD mouse model. (**A**) Representative images of H&E and Sirius red-stained WAT section and scoring of interstitial fibrosis of STD, HFD and HFD + PBI-4547 mice. (**B**) Evaluation of adipocyte maximum diameter and adipocyte surface area. Data are presented as mean ± SEM (n ≥ 6 per group, one-way ANOVA with Dunnett’s multicomparison test vs HFD). (**C**) Relative mRNA expression levels of genes related to fibrosis, inflammation, browning/thermogenesis, adipokines and glucose transporters. Geometric mean expression of qRT-PCR data was set to 1 for HFD group.

Since PBI-4547 modulated adipokine-related gene expression in the liver, we also evaluated the effects of this compound on white adipose tissue (WAT). In WAT, PBI-4547 decreased immune cell infiltration and the extent of interstitial fibrosis. (Fig. 5A). However morphometric analysis revealed that PBI-4547 did not affect the maximum diameter or average size of adipocytes (Fig. 5B). mRNA expression levels of several pro-fibrotic (*Cola1*, *Ctgf, Mmp2* and *Timp1*) and pro-inflammatory (*Il6* and *Ccl2*) genes were also downregulated by PBI-4547 (Fig. 5C). Additionally, PBI-4547 induced expression of *Pgc1a* and *Ucp1*, two genes intimately involved in thermogenesis and adipose tissue browning. Finally, expression of adipokine-related vaspin (*Serpina12)* was completely abolished by PBI-4547 while resistin and glucose transporters *Slc2a2* and *Slc2a4* were increased.

### PBI-4547’s mechanism of action differs from thiazolidinediones

We next sought to confirm our above findings obtained from the diet-induced model of obesity by using the *ob/ob* leptin-deficient genetic mouse model of metabolic syndrome. Additionally, since PBI-4547 was shown to bind and activate PPARγ, we compared the effects of this compound to pioglitazone, a clinically approved thiazolidinedione and known PPARγ agonist. Histological analysis of liver sections revealed that PBI-4547 was more effective than pioglitazone in preventing the clinical manifestations of fatty liver disease (Fig. S4A,B). While mice treated with pioglitazone had an increased trend towards weight gain, PBI-4547 significantly reduced bodyweight in *ob/ob* mice (Fig. S4C). Additionally, the mRNA expression profiles of glucose- and FA-related genes in liver (Fig. S4D,E) and WAT (Fig. S5) also differed between pioglitazone- and PBI-4547-treated mice.

### GPR84 plays a crucial role in glucose metabolism and the glucose-sensitizing effects of PBI-4547

**Figure 6:**
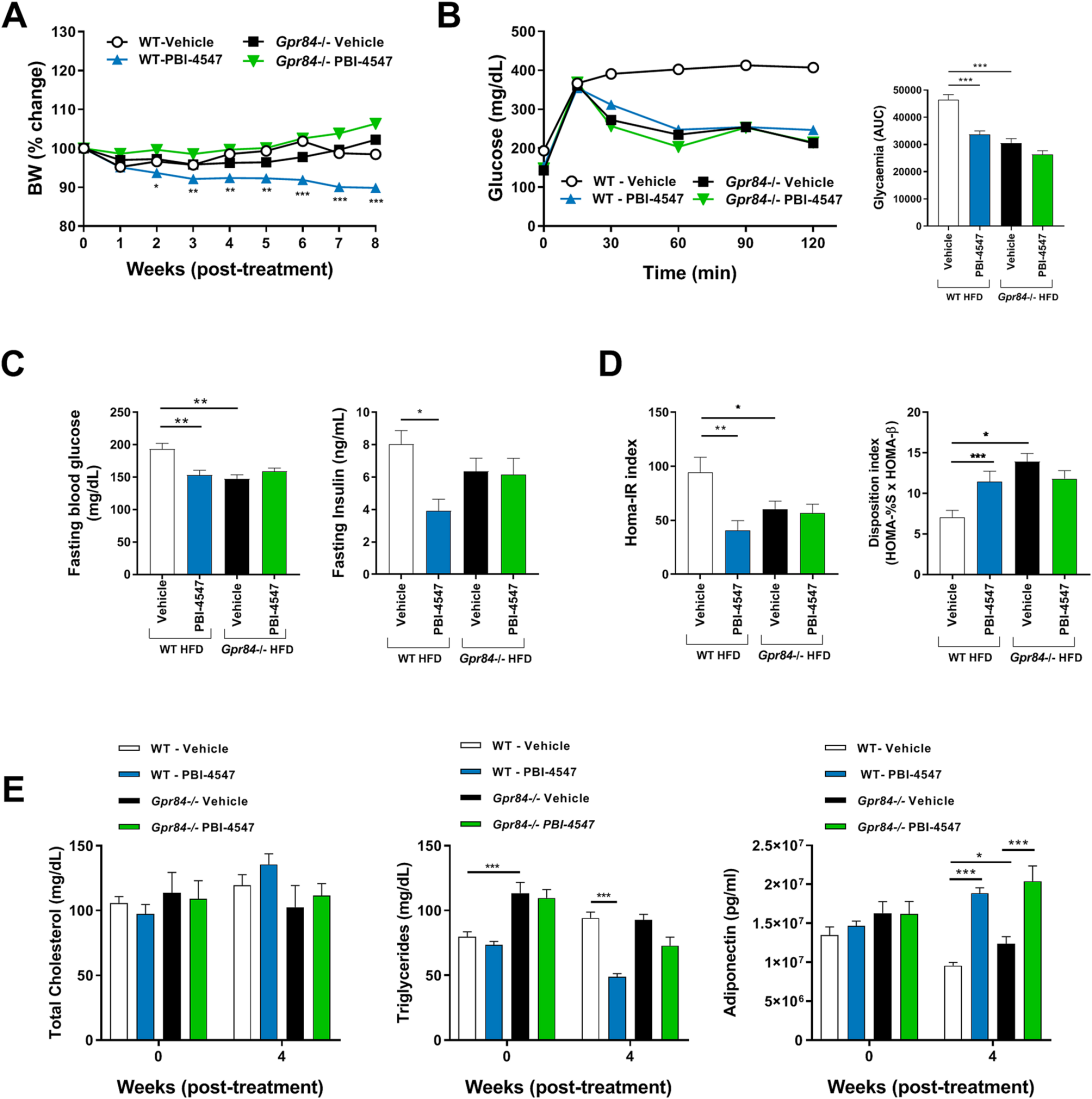
Assessment of glycemic parameters and insulin resistance in HFD-fed WT and *Gpr84*^*−/−*^ mice. (**A**) Average body weight (%) of mice 0–8 weeks after beginning of treatment. (**B**) Oral Glucose Tolerance Test (OGTT) and area under the curve of the OGTT of mice after 6 weeks of treatment. (**C**) Average fasting blood glucose and fasting insulin levels of the mice after 6 weeks of treatment. (**D**) Insulin resistance (HOMA-IR) and glucose disposition index of mice after 6 weeks of treatment. (**E**) Measurements of total cholesterol, triglycerides and adiponectin levels in serum of HFD mice after 0 and 4 weeks of treatment. Data are presented as mean ± SEM (n ≥ 9 per group, one-way ANOVA with Bonferroni’s multicomparison test).

Based on PBI-4547’s pharmacological profile and binding activities, we next used *Gpr84*^*−/−*^ mice fed a HFD to investigate the role of GPR84 in mediating the protective effects of PBI-4547 on glucose and FA metabolism. PBI-4547 decreased body weight in WT but not in HFD-fed *Gpr84*^*−/−*^ mice (Fig. 6A). Glucose was more rapidly metabolized in KO-mice compared to WT mice and PBI-4547 only had a significant effect on OGTT in WT mice (Fig. 6B). Moreover, fasting blood glucose levels were lower in KO compared to WT mice and were downregulated by PBI-4547 only in WT mice (Fig. 6C). Insulin levels and HOMA-IR index was reduced in WT but not in *Gpr84*^*−/−*^ mice treated with PBI-4547, confirming the glucose-sensitizing effect of PBI-4547 and the importance of GPR84 in mediating these effects (Fig. 6D). Untreated *Gpr84*^*−/−*^ mice HOMA-IR and disposition indices were also significantly improved compared to WT mice, suggesting that GPR84 is important in insulin resistance and in the control of glycemia, respectively. While cholesterol levels remained unchanged, triglyceride levels were lowered by PBI-4547, but once again only in WT mice (Fig. 6E). Interestingly, adiponectin was upregulated in both WT and *Gpr84*^*−/−*^ mice by PBI-4547, suggesting this effect may be GPR84-independant. Taken together, these data suggest PBI-4547 signals mainly via GPR84 to improve glucose handling.

### PBI-4547 mediates its protective effects via GPR84 in liver and WAT

**Figure 7:**
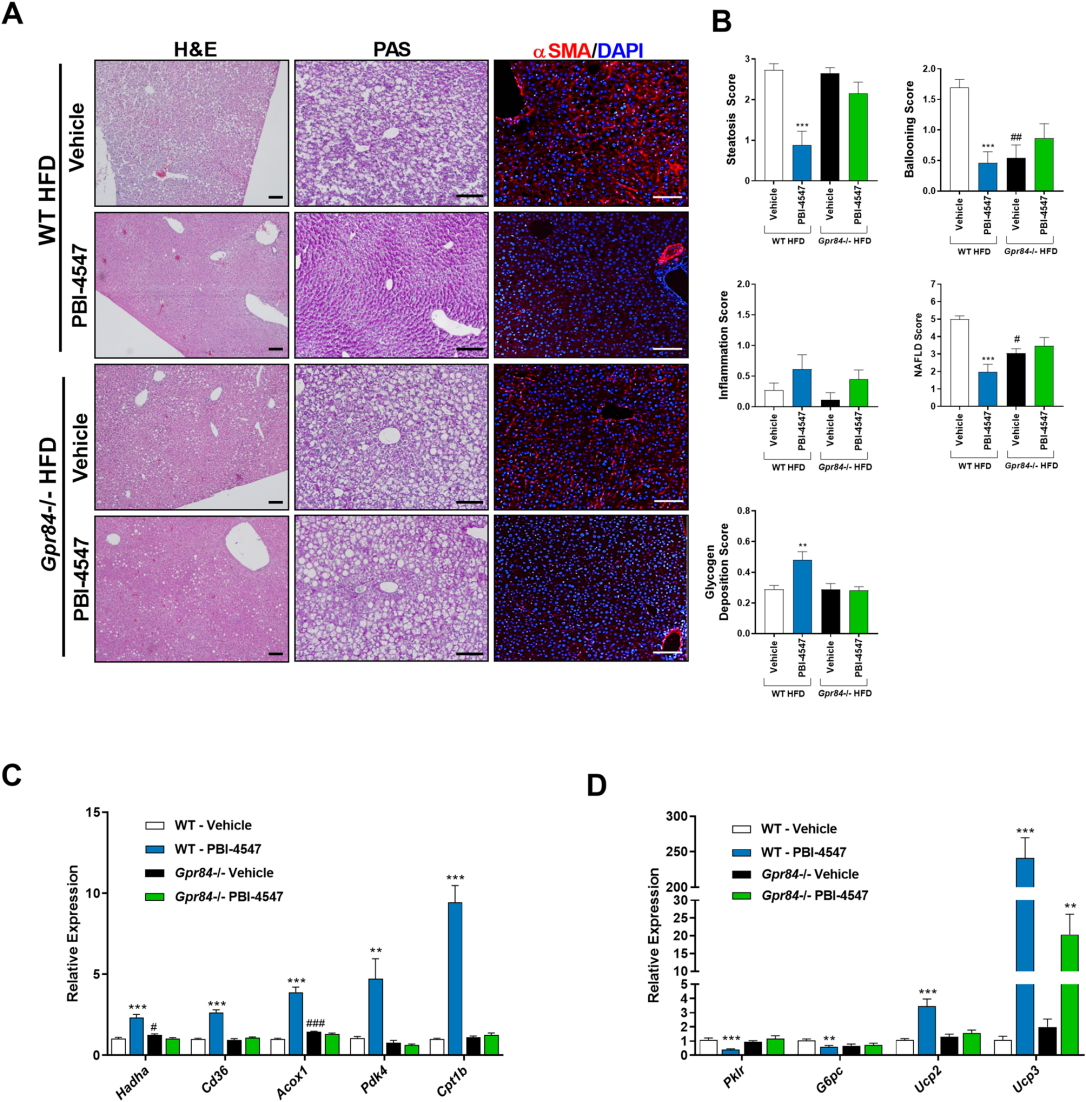
Effects of PBI-4547 intervention on NAFLD development in HFD-fed WT and *Gpr84*^*−/−*^ mice. (**A**) Representative images of H&E, PAS and α-SMA stained liver sections of WT and *Gpr84*^*−/−*^ mice. (**B**) Scoring of steatosis, ballooning, inflammation, total NAFLD score and glycogen deposition in liver sections of WT and *Gpr84*^*−/−*^ mice. Relative mRNA expression levels of (**C**) FA and (**D**) glucose-related genes in liver tissues of WT and *Gpr84*^*−/−*^ mice. Geometric mean expression of qRT-PCR data was set to 1 for WT vehicle group. Data are presented as geometric mean ± SEM ^#^P ≤ 0.05, ^##^P ≤ 0.01, ^###^P ≤ 0.001 between WT-vehicle and *Gpr84*^*−/−*^ vehicle (n ≥ 6 per group, one-way ANOVA with Bonferroni’s multicomparison test). Scale bar 100 μm.

Histological assessment of HFD-fed WT and KO mouse liver tissue revealed that *Gpr84*^*−/−*^ mice were more resistant to the development of obesity-induced NAFLD (Fig. 7A,B). Although the level of hepatic steatosis remained the same, ballooning was less evident in KO mice compared to WT mice. Furthermore, the impact of PBI-4547 on steatosis was highly dependent on GPR84 since this protective effect was lost in KO mice. Interestingly, PBI-4547 increased glycogen levels in WT mice but not in KO mice, suggesting an improvement in hepatocyte viability and energy storage capacity. To further characterize the impact of PBI-4547 in HFD-induced hepatic fibrosis, we measured immunodetectable alpha-SMA (α-SMA) by immunofluorescence staining. As shown in Fig. 7A, and corroborating our histopathological findings, diet-induced obesity was associated with increased immunodetectable α-SMA in liver sections, indicative of activated stellate cells. PBI-4547 treatment led to a dramatic drop in a-SMA positive liver staining. Moreover, in vehicle-treated HFD-fed GPR84^*−/−*^ mice, α-SMA staining was also present, however to a lesser degree than in WT mice. Of interest, PBI-4547 treatment in GPR84^*−/−*^ mice further decreased immunodetectable α-SMA, suggesting a possible off target effect in this regard. Expression of hepatic genes previously found to be regulated by PBI-4547 in WT mice were no longer modulated in *Gpr84*^*−/−*^ mice (Fig. 7C,D). Of note, *Hadha* and *Acox1* were slightly more elevated in *Gpr84*^*−/−*^ mice compared to WT mice. However, WT and KO mice had similar hepatic triglyceride and cholesterol contents (data not shown).

**Figure 8:**
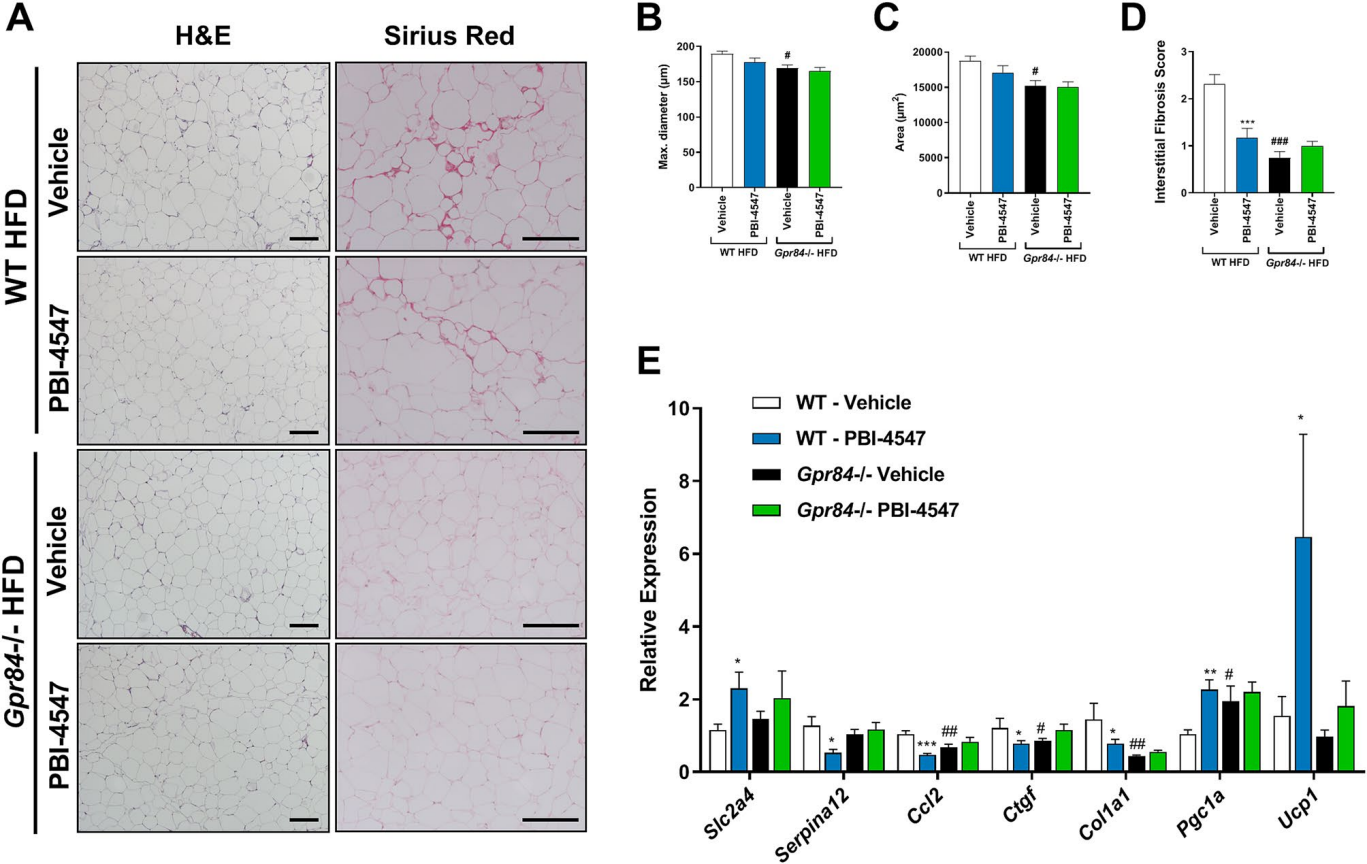
Effects of PBI-4547 intervention on WATs in HFD-fed WT and *Gpr84*^*−/−*^ mice. (**A**) Representative images of H&E and Sirius red stained WAT section of WT-vehicle, WT-PBI-4547, *Gpr84*^*−/−*^ vehicle and *Gpr84*^*−/−*^ PBI-4547 mice. Evaluation of (**B**) adipocyte maximum diameter (**C**) adipocyte surface area and (**D**) interstitial collagen deposition. (**E**) Relative mRNA expression levels of genes modulated by PBI-4547. Geometric mean expression of qRT-PCR data was set to 1 for WT vehicle group. Data are presented as Geometric mean ± SEM; ^#^P ≤ 0.05, ^##^P ≤ 0.01, ^###^P ≤ 0.001 between WT-vehicle and *Gpr84*^*−/−*^ vehicle (n ≥ 6 per group, one-way ANOVA with Bonferroni’s multicomparison test). Scale bar 250 μm.

The effects of PBI-4547 on *Gpr84*^*−/−*^ mice was also examined in WAT (Fig. 8). Both mean adipocyte diameters and area remained unchanged by PBI-4547. However, *Gpr84*^*−/−*^ mice had reduced adipocyte size and interstitial fibrosis compared to WT mice. Pro-inflammatory (*Ccl2)* and pro-fibrotic (*Ctgf and Col1a1)* gene expression were reduced while *Pgc1a* was increased in WAT from *Gpr84*^*−/−*^ mice. As observed in liver, PBI-4547’s modulatory effect on pro-inflammatory and pro-fibrotic markers was absent in WAT of mice lacking the GPR84 receptor. Collectively, these results indicate that PBI-4547 mediates its beneficial effects on hepatic and adipose tissue injury, mainly through binding and antagonism of GPR84.

## Discussion

Free fatty acid receptors have emerged as potential targets for the treatment of diabetes and metabolic syndrome^13–15^. In fact, GPR40 agonists have been used for the treatment of diabetes in clinical trials but have shown conflicting results^6,13^. Although GPR84 has been associated with inflammation in many reports^16–19^, studies evaluating the role of GPR84 in glucose and FA metabolism have been quite limited^20^. In this study, PBI-4547, a fatty acid mimetic, was used as a tool to study the role of GPR84 in glucose/FA metabolism and in NAFLD development. Our results were also validated using *Gpr84*^*−/−*^ KO mice. Our data indicate that GPR84 plays a crucial role in glucose and FA metabolism. Indeed, glucose levels and insulin resistance index were significantly decreased while the disposition index, indicative of β-cell function and its capacity to compensate during insulin resistance, was increased in *Gpr84*^*−/−*^ mice compared to WT mice. These results suggest that GPR84 is an important player in the regulation of glucose metabolism and possibly in β-cell physiology and function. Furthermore, based on liver histology, hepatocellular ballooning was in part dependent on GPR84 signaling as untreated GPR84-null mice showed decreased levels of ballooning compared to WT counterparts. Although PBI-4547 upregulated UCP’s, adiponectin and stimulated FAO, its effect on hepatic steatosis seems independent of GPR84 since KO mice developed similar levels of steatosis upon HFD-feeding. Therefore, the mechanism underlying the progression from steatosis to ballooning maybe driven in part by GPR84 activation. In accordance with these results, triglyceride levels were significantly lower in *Gpr84*^*−/−*^ mice.

Consumption of a hypercaloric diet leads to increased levels of circulating FAs which are in part taken-up by the liver. In addition, accumulation of fatty acids, one of the hallmarks of NAFLD, can occur through increased FA uptake and/or impaired FAO^21,22^. High concentrations of circulating FAs lead to lipotoxicity and compromised glucose uptake, thereby promoting the development of insulin resistance^23^. Our results demonstrate that PBI-4547 increases FAO leading to reduced steatosis, ballooning and NAFLD score. PBI-4547 increased the expression of several enzymes involved in FAO, including acyl-CoA dehydrogenase 1 (*Acox1*) and hydroxy-acyl-CoA dehydrogenase (*Hadha*), the first and third enzymes involved in the FAO pathway. Next, Cd36 and Cpt1, responsible for FA uptake, were also upregulated by PBI-4547. Finally, *Pdk4* which inhibits the pyruvate dehydrogenase complex (PDC), thereby promoting utilization of FA’s, was also increased by PBI-4547. Moreover, defective FAO has also been linked to the development of fibrosis^24,25^. In this study, PBI-4547 was shown to reduce the expression of several pro-fibrogenic genes in both liver and WAT, including *Col1a1*, *Ctgf* and *Mmp2*. Interestingly, pharmacological restoration of FAO prevented cell damage and completely abolished fibrosis in a mouse model of renal fibrosis^26^ suggesting that augmentation of FAO by PBI-4547 could prevent fibrosis and thus NAFLD progression.

Recent reports indicate that defects in both glycolysis and β-oxidation are associated with various pathologies. Indeed, the metabolic unbalance between glucose and FA utilization is associated with various kidney and liver pathologies^4,26,27^. This metabolic reprogramming is linked to mitochondrial dysfunction and pharmacological restoration of adequate energy metabolism is currently under investigation for various indications, including diabetes, NAFLD and chronic kidney diseases (reviewed in Refs.^4,28,29^. Our results suggest that PBI-4547 restores the proper balance between FA and glucose utilization in an HFD mouse model. Moreover, altered amino acid concentrations and Krebs’s cycle intermediates, which are known to be dysregulated in obesity, T2D patients and NAFLD^30,31^, were also restored to normal levels by PBI-4547. PBI-4547 also restored high levels of NAD + in liver. Interestingly, NAD + repletion has been linked with improved mitochondrial and stem cell function and increased rodent lifespan^32^.

Our findings found that PBI-4547 improved insulin resistance. Indeed, fasting insulin levels and glycemia were lowered after drug treatment. PBI-4547 also decreased liver expression of *G6pc*, suggesting a possible involvement in gluconeogenesis, thereby contributing to reduction of glycemia. WAT, which also plays a central role in the regulation of glycemia, was likewise positively affected by PBI-4547. Of note, glucose transporters GLUT2 and GLUT4 were both increased in WAT from PBI-4547-treated mice. The regulation of these factors by PBI-4547 is likely to contribute to WAT homeostasis and increased adiponectin secretion. To this end, increased adiponectin secretion in turn, is largely associated with activation of several hepatic pathways involved in fatty acid oxidation, reduced lipid synthesis and prevention of steatosis^33,34^. These results suggest that PBI-4547 may regulate crosstalk between liver and WAT under pro-inflammatory and/or conditions of metabolic dysregulation. Moreover, this crosstalk could be largely attributed to GPR84 as PBI-4547 lost most of its effects in KO mice. In addition, WAT from KO mice had very limited amounts of interstitial fibrosis and immune cell infiltration in HFD-fed mice, accompanied by decreased levels of pro-inflammatory and pro-fibrotic markers, indicating that GPR84 is directly involved in WAT inflammation and fibrosis in this context.

Mitochondrial uncoupling agents promote augmentation of energy expenditure that can be increased through stimulation of UCPs^35–37^. A recent report indicates that promoting liver-specific UCP expression reverses hypertriglyceridemia, NAFLD and insulin resistance, indicating that UCPs play a crucial role in glucose and FA metabolism^38^. Another study by the same group indicates that 2–4 dinitrophenol, a mild mitochondrial uncoupling agent, reverses diabetes and steatohepatitis^39^. These reports are in accordance with data presented in our study indicating that PBI-4547 increases *Ucp1* in WAT and *Ucp-2/3* in liver, promoting mitochondrial proton leak and increasing overall energy expenditure. Moreover, increased UCP1 expression in WAT and increased levels of serum adiponectin from PBI-4547-treated mice correlate with a larger brown adipose tissue fat pad volume (M.T., Lil. G., M.P.C., K.H., S.L., personal communication). OCR experiments confirmed that PBI-4547 induces proton leak in hepatocytes through the mitochondrial intermembrane space. These results are likely to explain the effect of PBI-4547 on energy metabolism and NAFLD development. It is noteworthy that dozens of drugs are reported to shift energy metabolism and to inhibit mitochondrial respiration^40^. Among these drugs, the anti-diabetic drug metformin was reported to decrease OCR. Metformin inhibits multiple targets, including adenylate cyclase and NADH:ubiquinone oxidoreductase, thereby leading to reduced levels of ATP and decreased levels of oxygen consumption. Metformin also mediates its effects through phosphorylation and activation of AMPK. In a similar fashion, PBI-4547 decreased ATP production and maximal respiration. PBI-4547 has also shown to promote AMPK phosphorylation levels in the liver (data not shown).

The effect of PBI-4547 on adiponectin secretion was independent of GPR84 as *Gpr84*^*−/−*^ mice treated with PBI-4547 also had high levels of adiponectin. Our results also show that steatosis establishment was independent of GPR84 suggesting that other receptors than GPR84 that are targeted by PBI-4547, such as GPR40 or PPARs, may be involved in adiponectin secretion and the process of steatosis. Interestingly, PPARγ ligands are known to increase serum adiponectin concentrations^41^^,^ and hence activation of this receptor may be implicated in this effect of PBI-4547. Moreover, all three PPARs are activated by fatty acids or fatty acid derivatives and regulate lipid metabolism and inflammation^42,43^. PPARα was previously shown to mediate fatty acid oxidation in liver, while PPARγ is linked to insulin resistance and lipid storage in adipocytes. Stimulation of PPARs might also contribute to UCP expression as a PPARα agonist induced UCP2 in rodent hepatocytes^44^. PPARγ stimulation also induced UCP2 expression in both in vitro and in vivo studies^45–47^. It is thus likely that increased UCP expression in PBI-4547-treated mice could be attributed to PPAR activity. Therefore, PPARs could be directly or indirectly involved in GPR84 signaling. However, in the context of the measured parameters in our model, the activity of PBI-4547 on these other receptors seemed limited, as besides the adiponectin increase and a residual increase of liver *Ucp3* expression, the effects of PBI-4547 were eliminated in the absence of GPR84.

In summary, we have demonstrated that GPR84 is a new important regulator of FA and glucose metabolism. Interestingly, GPR84 was also shown to be crucial for the transition from steatosis to ballooning in NAFLD. PBI-4547, mainly through its antagonistic effects on GPR84 activity, provided protection against NAFLD and diabetes, highlighting the potential of this drug in this rapidly growing indication.

## Methods

### Chemicals

PBI-4547 is a low molecular weight orally active chemical entity designed as a mimetic of medium-chain fatty acids. PBI-4547 was synthesized by Liminal R&D Biosciences Inc. (Laval, Qc, Canada) in five steps using Suzuki coupling, as follows: 2-[3,5-dibromophenyl]acetic acid was converted to the corresponding ester and then reacted with pent-1-enylboronic ester in the presence of palladium catalyst to give 3,5-dipent-1-ene derivative. This compound was hydrogenated over palladium, hydrolyzed to the acid, and treated with a base to yield the expected product PBI-4547.

### Cell culture and transfection

Human embryonic kidney (HEK) 293 cells (Sigma-Aldrich) were cultured in Eagle’s minimum essential medium (Wisent, Saint-Jean-Baptiste, QC, Canada) supplemented with 2 mmol/L l-glutamine, 10% FBS (Wisent), and 1% nonessential amino acids (Sigma-Aldrich). Transient HEK293 transfections were performed using polyethylenimine (Polysciences, Warrington, PA), as described previously^8,48^. Hepatocellular carcinoma HepG2 cells were cultured at 37 °C 5% CO_2_ in EMEM supplemented with 10% FBS, 10 mM sodium pyruvate and non-essential amino acids.

### Plasmids

Generation of the Gα_i2_, Gα_q_, and GPR40/β-arrestin-2 BRET biosensors have been described previously^8^. A plasmid encoding the human GPR120-L (long isoform) cDNA was obtained from R&D Systems. GPR120-S (short isoform) was generated by replacing the BglII-BsgI fragment from GPR120-L by a gBlock gene fragment (Integrated DNA Technologies, Coralville, IA) lacking the DNA sequence corresponding to the extra 16 amino acids found in the third intracellular loop of the long form. The GPR120-GFP10 construct was generated by inserting PCR-amplified GPR120-S (without its stop codon) in place of GPR40 in the GPR40-GFP10 plasmid. The hinge region and ligand binding domain (LBD) from human PPARα (S167–Y468), PPARδ (S139–Y440) and PPARγ (S176–Y477) were PCR-amplified from a PPARα cDNA clone (cDNA Resource Center, https://www.cdna.org) or from PPARδ1 and PPARγ1 LBD gBlocks gene fragments. The PPAR LBD PCR products were inserted in frame with the GAL4 DNA binding domain in the pFN26A(BIND)-hRluc-neo Flexi vector (Promega) at SgfI and PmeI sites. The pGL4.35[luc2P/9XGAL4UAS/Hygro] vector was obtained from Promega. All generated constructs were verified by DNA sequencing.

### BRET measurement

BRET measurements in transiently transfected HEK293 cells were performed as previously described^8^. Ligands were incubated with cells at room temperature for 10 min (G protein biosensors) or 25 min (β-arrestin/GPR120) before collecting BRET^2^ readings between Rluc8 and GFP10.

### TR-FRET PPAR Competitive binding assay

The LanthaScreen time-resolved fluorescence resonance energy transfer (TR-FRET) PPARα, PPARδ and PPARγ competitive binding assay (Fisher Scientific) was performed according to the manufacturer’s protocol. Compounds were incubated with glutathione S-transferase (GST)-tagged human PPAR-LBD, terbium-labeled anti-GST antibody, and a fluorescent small molecule pan-PPAR tracer ligand (Fluormone pan-PPAR Green) for 4 h in the dark at room temperature. The FRET signal was measured using an Infinite M1000 microplate reader by excitation at 332 nm and emission at 520 nm for fluorescein and 490 nm for terbium. Competitive ligand binding to PPAR is detected by a test compound’s ability to displace the Fluormone pan-PPAR Green from the LBD, which results in a decrease of the 520 nm/490 nm TR-FRET ratio. The inhibition constant (K_i_) of the competitor ligand was calculated by applying the Cheng-Prusoff equation.

### Cell-based transactivation assay

HEK293 cells were co-transfected with pGL4.35[luc2P/9XGAL4UAS/Hygro] and GAL4-PPAR-Rluc plasmids, and after 24 h of incubation were treated with compounds for 24 h. Luciferase activity was determined with the Dual Glo luciferase assay (Promega). Firefly luminescence was normalized to the constitutively expressed *Renilla* luminescence, and results expressed as fold induction of vehicle control.

### Hepatic cholesterol and triglyceride quantification

Total cholesterol levels were measured using the HDL/LDL/VLDL Cholesterol Assay kit (Abcam) according to manufacturer’s protocol. Results were reported as mg of total cholesterol per mg of liver tissue. Liver triglycerides were extracted using a modified version of the Folch protocol^49^^,^ then quantified using a triglyceride colorimetric kit (Cayman Chemical).

### High-fat diet mouse model

WT or *Gpr84*^*−/−*^ mice, as previously described, (aged 10–14 weeks at the beginning of the experiment) were either fed a standard diet (STD) or a high-fat diet (HFD) (Envigo #TD.06414) for 14 weeks. At this point, mice were administered either vehicle (water) or PBI-4547 (10 mg/kg) on a daily basis for 6 (WT study) or 8 (WT vs KO study) weeks by gastric gavage, and were sacrificed thereafter. Liver and WAT tissues were fixed in 10% formalin for histological analyses or flash frozen in liquid nitrogen for RT-PCR analyses. All animal studies were reviewed and approved by the animal care and use committee of the Institut National de la Recherche Scientifique – Armand-Frappier (Laval, QC, Canada) and the University of Ottawa’s Animal Care and Veterinary Services (Ottawa, ON, Canada). Protocols were approved and undertaken according to the Canadian Council on Animal Care guidelines.

### Oral glucose tolerance test

Oral glucose tolerance tests (OGTT) were completed 6 weeks after beginning of the treatments. Briefly, mice were fasted for 4–6 h, given a bolus of glucose at 2 mg/kg BW orally, and blood glucose values measured at 0, 15, 30, 60, 120 min. In parallel, a blood sample was obtained at 0, 15, 60 and 120 min for insulin quantification by ELISA (EMD Millipore).

### Histology and immunofluorescence

After fixation in formalin, iWAT (inguinal), eWAT (epididymal) and liver sections were embedded in paraffin. Thin section of 5 μM were stained with Hematoxylin and Eosin or Sirius red for analyses. WAT collagen deposition and liver characteristics of NAFLD were quantified histologically by a pathologist (R.C.) in a double-blinded manner. Liver histology was performed on H&E stained slides. WAT collagen deposition was scored using Picro Sirius-Red stained slides. Immunofluorescence-detection of α-SMA in liver sections was performed. Briefly, samples were deparaffinized in a series of xylenes, rehydrated in graded alcohols followed by an antigen-unmasking step by microwaving in Tris-buffer (pH 8.2). Sections were blocked with 10% donkey serum (Vector labs), 1% bovine serum albumin (Sigma) in PBS + 1%Tween for 1 h at room temperature. Slides were incubated overnight at 4 °C with an anti-mouse smooth muscle α-Actin antibody (1:300; Santa Cruz, clone #1A4). Secondary detection was performed using a donkey anti-mouse Alexa Fluor 594 (1:500; Molecular Probes), incubated for 1 h at room temperature. Slides were dehydrated and mounted with fluorescent antifade media containing DAPI (Vector labs). Images were taken at 100X and 200X magnification and representative images are shown.

### Gene expression

RNA was extracted and purified as previously described^8^. Real-time quantitative PCR was performed on a CFX96 Touch Real-Time PCR Detection System (Bio-Rad) using TaqMan gene expression assays (Fisher Scientific). qPCR data were analyzed using the 2-^ΔΔCt^ method, using *Gapdh* and *Hprt* as endogenous control. RT-qPCR data were reported as Geometric means ± SEM.

### OCR and ECAR experiments

Oxygen consumption rate (OCR) and extracellular acidification rate (ECAR) were measured with a Seahorse XF96 as per manufacturer’s protocol (Agilent). Briefly, 8 × 10^4^ HepG2 cells were plated onto Seahorse XF96 96-well plates for 24 h prior to stimulation. The next day, cells were treated with PBI-4547 or with vehicle control for 24 h. Cells were subjected to a mitochondrial stress test with serial injections of Oligomycin (1 μM; ATPase inhibitor), FCCP (1 μM; mitochondrial uncoupler) and Rotenenone/Antimycin A (1 μM; complex I/III inhibitor).

### ^1^H-NMR liver metabolomics profiling

A portion of hepatic tissue (50 mg) was mechanically homogenized in 1 mL H_2_O/CH_3_CN (1/1) using a PowerGen 125 homogenizer (Fisher Scientific). The homogenates were centrifuged for 10 min at 5,000×*g* at 4 °C. Hydrophilic metabolites were lyophilized to remove water for NMR experiments. The metabolites were reconstituted in 200 μL D_2_O containing 100 µM 3-(Trimethylsilyl)propionic-2,2,3,3-d4 acid (TSP) as reference standard. Extracts were vortexed for 20 min, centrifuge at 5,000×*g* for 10 min and transferred into 1.7 mm-NMR glass tubes.^1^H-NMR spectra were acquired using a Bruker Avance III 600 MHz. Resonance assignments were based on the literature values and HMDB database. Standard solutions of metabolites were used to confirm peak assignments. NMR spectra were integrated to obtain relative concentrations derived from liver extracts.

### Statistical analyses

Data are expressed as means ± SEM for each treatment compared with vehicle control. Statistical analysis was performed using either one-way analysis of variance (when appropriate) with Tukey’s, Dunnett’s or Bonferroni post-test for multiple comparisons or t-test (two tailed) when comparing two groups. All data were analyzed using GraphPad Prism version 7 for Windows (GraphPad, San Diego, CA). Statistical significance was set as followed: *P ≤ 0.05; **P ≤ 0.01; ***P ≤ 0.001.

## Supplementary information

Supplementary information
