# Supplementary material for: Fatty acid mimetic PBI-4547 restores metabolic homeostasis via GPR84 in mice with non-alcoholic fatty liver disease
Source: Sci Rep. 2020 Jul 29;10:12778. doi: 10.1038/s41598-020-69675-8 (PMC7391726; doi:10.1038/s41598-020-69675-8)
Supplement: Supplementary file 1 — Supplementary information [file 41598_2020_69675_MOESM1_ESM.docx]

**Fatty Acid Mimetic PBI-4547 Restores Metabolic Homeostasis via GPR84 in Mice with Non-**

**Alcoholic Fatty Liver Disease**

Jean-Christophe Simard^1^*, Jean-François Thibodeau^1,2^* Martin Leduc^1^, Mikael Tremblay^1^, Alexandre Laverdure^1^, François Sarra-Bournet^1^, William Gagnon^1^, Jugurtha Ouboudinar^1^, Liette Gervais^1^, Alexandra Felton^1^, Sylvie Letourneau^1^, Lilianne Geerts^1^, Marie-Pier Cloutier^1^, Kathy Hince^1^, Ramon Corpuz^1^, Alexandra Blais^1^, Vanessa Marques Quintela^1^, Jean-Simon Duceppe^1^, Shaun D. Abbott^1^, Amélie Blais^2^, Boulos Zacharie^1^, Pierre Laurin^1^, Steven R. Laplante^3^, Christopher R.J. Kennedy^2^, Richard L. Hébert^2^, François A. Leblond^1^, Brigitte Grouix^1^ and Lyne Gagnon^1^

1. Liminal R&D Biosciences Inc., 500 Boul. Cartier Ouest, Laval, Québec, Canada. H7V 5B7
2. Kidney Research Centre, Ottawa Hospital Research Institute / Department of Cellular and Molecular Medicine, University of Ottawa, 451 Smyth Road, Ottawa, Ontario, Canada. K1H 8M5
3. Institut National de la Recherche Scientifique, Institut Armand-Frappier, 531 Boul. Des Prairies, Laval, Québec, Canada. H7V 5B7

*These authors contributed equally to the work

**Supporting Information**

**Table S1. Endpoint parameters in PBI-4547 treated standard diet-fed mice**

|  | STD | | |  | STD+4547 | | |  |
| --- | --- | --- | --- | --- | --- | --- | --- | --- |
|  | Mean | SEM | n |  | Mean | SEM | n | P-value |
| Bodyweight  *(g)* | 35.59 | 1.1 | 10 |  | 35.59 | 1.0 | 10 | >0.99 |
| Food intake, *(g.mouse^-1^.wk^-1^)* | 21.73 | 0.7 | 6 |  | 20.95 | 0.8 | 6 | 0.48 |
| Glucose tolerance,  *(AUC)* | 1492.60 | 47.9 | 10 |  | 1412.00 | 35.8 | 10 | 0.19 |
| Fasting glucose, *(mM)* | 11.16 | 0.5 | 10 |  | 11.58 | 0.4 | 10 | 0.53 |
| Fasting insulin, *(ng/mL)* | 0.76 | 0.2 | 9 |  | 0.94 | 0.4 | 9 | 0.69 |
| HOMA-IR  *(Index)* | 7.83 | 2.1 | 9 |  | 7.74 | 3.2 | 10 | 0.98 |
| HOMA-β  *(Index)* | 79.88 | 23.6 | 9 |  | 116.96 | 60.3 | 10 | 0.59 |

**Figure S1. Effects of PBI-4547 on organ weight and food intake in normal and HFD-fed mice mouse model.** Measurements of (**A**) Average food consumption. (**B**) WAT, (**C**) liver, (**D**) pancreas and (**E**) kidney weight.

**Figure S2. Effects of PBI-4547 on collagen deposition in a mouse model of CCL4 and a rat model of BDL. (A)** Masson’s Trichrome staining in liver section from a CCL4 mouse model. (B) Picro Sirius staining in liver section from a BDL rat model. Graphs represent collagen quantification. Data are presented as mean ± SEM; * P ≤ 0.05, ** P ≤ 0.01, ***P ≤ 0.001 (n ≥ 6 per group, one-way ANOVA with Dunnett’s multicomparison test, CCL4 or BDL are reference group)

**Figure S3. Metabolic profiling in liver from a HFD mouse model.** Relative ^1^H NMR quantification of (A) amino acids, (B) Krebs cycle intermediates, (C) Glucose/energy-related metabolites and (D) other metabolites in liver from STD, HFD and HFD + PBI-4547 mice. Mean expression of data was set to 1 for HFD group. Data are presented as mean ± SEM (n ≥ 4 per group, one-way ANOVA with Dunnett’s multicomparison test vs HFD)

**Figure S4. Comparative effects of PBI-4547 and Pioglitazone on the metabolism of ob/ob mice.** (**A**) Representative image of H&E-stained liver section of ob/ob mice. (**B**) Evaluation of hepatic steatosis, ballooning, inflammation and total NAFLD score. Data are presented as mean ± SEM (n ≥ 6 per group, one-way ANOVA with Dunnett’s multicomparison test vs ctrl group). (**C**) Body weight of ob/ob mice over 14 days of treatment. Relative mRNA expression of (**D**) glucose and (**E**) FA-related genes. Geometric mean expression of qRT-PCR data was set to 1 for ctrl group.

**Figure S5. Comparative effects of PBI-4547 and Pioglitazone on WAT gene expression in ob/ob mice.** Relative mRNA expression of (**A**) FA and (**B**) thermogenesis/browning-related genes. Geometric mean expression of qRT-PCR data was set to 1 for Ctrl group. Data are presented as Geometric mean ± SEM (n ≥ 6 per group, one-way ANOVA with Dunnett’s multicomparison test vs ctrl group)

**Supporting Methods**

**Ob/ob mouse model**

Commercially available 6 week old leptin-deficient ob/ob mice were orally given PBI-4547 (50 mg/kg), Pioglitazone (20 mg/kg) or the vehicle for 14 days. Mice were then euthanized and tissues were harvested as mentioned previously.

**CCl_4_-induced liver fibrosis**

Liver fibrosis was induced in 6-weeks old male C57BL/6 mice as previously described^50^, briefly by an intraperitoneal (i.p.) administration of 2 ml/kg of CCl_4,_ prepared as a 10% solution in olive oil, twice a week for 58 days. Next, mice were randomly allocated to 4 groups. Sham group was injected with an equal volume of olive oil (i.p.) and orally administered an equal volume of distilled water. The CCl_4_ group was injected by i.p. with CCl_4_ and administered an equal volume of vehicle (distilled water) or PBI-4547 at 50 mg/kg (p.o) from day 1 to day 58 in a separate CCl_4_-treated group. Mice were sacrificed at day 59. Livers were collected to evaluate collagen deposition.

**Bile-Duct Ligation-induced fibrosis**

Liver fibrosis was induced following a double ligation of the common bile duct (BDL) in male Wistar rats by abdominal laparotomy under isoflurane anesthesia, as previously described^50^. Briefly, silk sutures were tied around the isolated BDL at both cranial and caudal ends and the BDL was transected between the ligatures. PBI-4547 at a dose of 50 mg/kg was orally administered between days 1 to 7. Rats were sacrificed at day 7 and liver tissue was collected for evaluation of collagen deposition.
